# A Comparative Study of ChatGPT and BingAI in Answering the National Eligibility Entrance Test for Postgraduates (NEET-PG)-Style Practice Questions: A Cross-Sectional Analysis

**DOI:** 10.7759/cureus.76108

**Published:** 2024-12-20

**Authors:** Subhav Ramnani, Manik Bhalla, Radhika Bassi

**Affiliations:** 1 Internal Medicine, Jaipur Golden Hospital, New Delhi, IND; 2 General Surgery, Holy Family Hospital, New Delhi, IND; 3 Internal Medicine, Ross University School of Medicine, Saint Michael, BRB

**Keywords:** artificial intelligence, comparative study, language models, medical education, neet-pg, plab

## Abstract

Purpose: The integration of artificial intelligence (AI) into medical education has witnessed significant progress, particularly in the domain of language models. This study focuses on assessing the performance of two notable language models, ChatGPT and BingAI Precise, in answering the National Eligibility Entrance Test for Postgraduates (NEET-PG)-style practice questions, simulating medical exam formats.

Methods: A cross-sectional study conducted in June 2023 involved assessing ChatGPT and BingAI Precise using three sets of NEET-PG practice exams, comprising 200 questions each. The questions were categorized by difficulty levels (easy, moderate, difficult), excluding those with images or tables. The AI models' responses were compared to reference answers provided by the Dr. Bhatia Medical Coaching Institute (DBMCI). Statistical analysis was employed to evaluate accuracy, coherence, and overall performance across different difficulty levels.

Results: In the analysis of 600 questions across three test sets, both ChatGPT and BingAI demonstrated competence in answering NEET-PG style questions, achieving passing scores. However, BingAI consistently outperformed ChatGPT, exhibiting higher accuracy rates across all three question banks. The statistical comparison indicated significant differences in correct answer rates between the two models.

Conclusions: The study concludes that both ChatGPT and BingAI have the potential to serve as effective study aids for medical licensing exams. While both models showed competence in answering questions, BingAI consistently outperformed ChatGPT, suggesting its higher accuracy rates. Future improvements, including enhanced image interpretation, could further establish these large language models (LLMs) as valuable tools in both educational and clinical settings.

## Introduction

Over the past decade, the rapid evolution of artificial intelligence (AI) has ignited discussions about its integration into medicine and education. AI's capacity to automate tasks, employ self-learning algorithms, and achieve accuracy with deep neural networks is particularly impactful in the field of medical imaging [1]. One noteworthy application is ChatGPT, a substantial language model developed by OpenAI. Utilizing self-attention mechanisms and extensive training data, ChatGPT generates contextually relevant responses in conversations. Its unique feature lies in operating as a server-contained model without internet access, crafting responses based on internal relationships between words in its neural network [2].

An examination of ChatGPT's performance on the United States Medical Licensing Examination (USMLE) revealed its effectiveness, achieving over 50% accuracy across all exams and reaching 60% in most analyses, closely aligning with the passing threshold [3]. In a parallel study on arXiv, another large language model, Flan-PaLM, achieved a notable 67.6% accuracy on the USMLE, surpassing prior benchmarks by 17 percentage points. Researchers emphasized the potential of large language models in advancing medical AI and enhancing accessibility, security, and equity. However, the integration of AI programs, including ChatGPT, in medical research papers has triggered discussions and concerns among healthcare professionals, as highlighted in a recent Nature article [4].

The National Eligibility Entrance Test for Postgraduates (NEET-PG) serves as an Indian national exam for medical postgraduate courses and is specific for admissions in India [5]. Due to the deficit in the literature pertaining to AI’s capabilities in Indian entrance examinations, the study aimed to assess chatbots and their efficiency in answering NEET-PG questions.

Aims and Objectives

The present study aimed to evaluate the responses of ChatGPT 3.5 and BingAI to NEET-PG style questions and to assess the accuracy, coherence, and overall performance of these AI systems across different difficulty levels.

## Materials and methods

Study Design and Setting

An observational cross-sectional study was conducted in June 2023 to assess the performance of two AI software, ChatGPT and Microsoft Copilot (formerly BingAI, accessed in June 2023), in answering National Eligibility Entrance Test for Postgraduates (NEET-PG) practice exams for postgraduate students [6,7]. The study size of 600 questions (three NEET-PG practice exams with 200 questions each) was determined based on the practical availability of questions rather than a formal sample size calculation. This number was deemed sufficient to provide meaningful statistical analysis and generalizability, considering the standardized structure of NEET-PG question banks.

The study adhered to ethical guidelines, with the exclusion of human subjects, and written consent was obtained from Dr. Bhatia Medical Coaching Institute (DBMCI) [8]. DBMCI was chosen due to its reputation as a leading provider of standardized NEET-PG preparation materials with high validity and reliability in simulating real exam scenarios. Access to DBMCI was granted for 10 days starting June 6, 2023. Ethical approval was deemed exempt as the study involved no direct human interaction.

Study Tools

To ensure focused evaluation, questions were categorized based on difficulty levels: easy, moderate, and difficult. Questions containing images or tables, which may challenge AI interpretation, were excluded to maintain uniformity in the textual input provided to the AI. While AI systems have the capability to interpret visual data, their performance on such inputs may vary depending on specific pre-processing requirements not standardized across models.

The selected questions were systematically inputted into ChatGPT and Bing AI Precise, and their responses, along with explanations for each question, were meticulously documented in Excel (Microsoft® Corp., Redmond, WA). Demographic and clinical information related to the NEET-PG questions, such as topic categorization and difficulty levels, were recorded. The exposures were the NEET-PG practice questions, while outcome events included AI-generated correct and incorrect responses.

*Statistical and Data Analysis*

The study's analytical phase involved a statistical comparison of AI-generated answers against reference answers provided by DBMCI to assess accuracy and coherence. "Coherence" was defined as the logical consistency and clarity of AI-provided explanations and was evaluated using a structured rubric. Scores were assigned by two independent reviewers to ensure objectivity.

Quantitative variables, such as correctness rates, were analyzed. The grouping of questions into easy, moderate, and difficult categories followed historical performance data. Statistical methods included descriptive statistics, accuracy rates, coherence scores, and inferential testing. Differences in correct answer rates between ChatGPT and Bing AI Precise were assessed using chi-square tests, and effect sizes (e.g., Cohen’s d) and confidence intervals were calculated to provide practical significance in addition to p-values.

## Results

The study included three sets of NEET-PG practice exams, each comprising 200 questions. Of the total questions, 78 from Test A, 66 from Test B, and 69 from Test C were excluded based on predefined criteria. Reasons for exclusion included image-based questions, questions containing tables, and questions unanswered by ChatGPT, BingAI, or both.

The final analysis thus included 122 questions from Test A, 134 from Test B, and 131 from Test C. The participant flow is outlined in Table 1, detailing the numbers at each study stage, from potential eligibility to analysis. 

**Table 1:** Total number of questions included in the study and the reason for exclusion.

Variables	Test A	Test B	Test C
Questions	200	200	200
Excluded	78	66	69
Images or tables	63	55	56
Unanswered by ChatGPT	1	1	0
Unanswered by BingAI	11	6	10
Unanswered by both	3	4	3
Included	122	134	131

Across all tests, BingAI demonstrated higher accuracy rates than ChatGPT, achieving a passing score for NEET-PG. The total correct answers for BingAI (n=325, 83.9%) significantly exceeded those for ChatGPT (n=253; 60.7%) with a p-value of < 0.001 (Table 2).

**Table 2:** Number of questions correctly answered by ChatGPT and BingAI.

Tests	Questions included	ChatGPT	BingAI	p-value
Test A	122	74	99	<0.001
Test B	134	91	113	<0.001
Test C	131	88	113	<0.001
Total	387	253	325	<0.001

**Figure 1:**
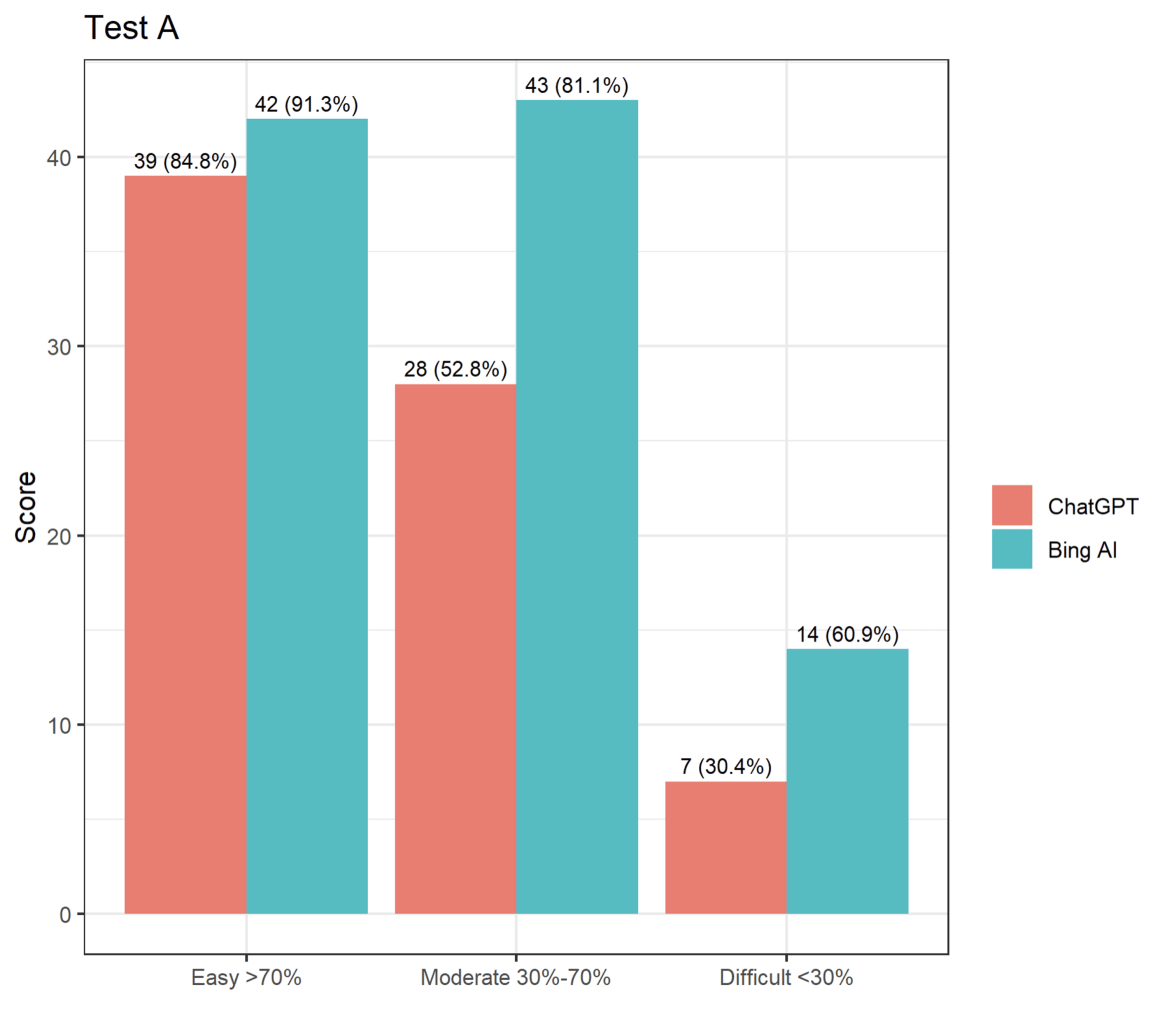
Comparison of answers by ChatGPT and BingAI for Test A.

In test A, BingAI had an accuracy rate of 81.14%, whereas ChatGPT had an accuracy rate of 60.65%, making BingAI approximately 20% more accurate than ChatGPT (Figure 1). BingAI consistently scored higher across all types of questions, with the highest difference observed for moderate questions, where BingAI scored 81.1% (n=43) and ChatGPT scored 52.8% (n=28).

**Figure 2:**
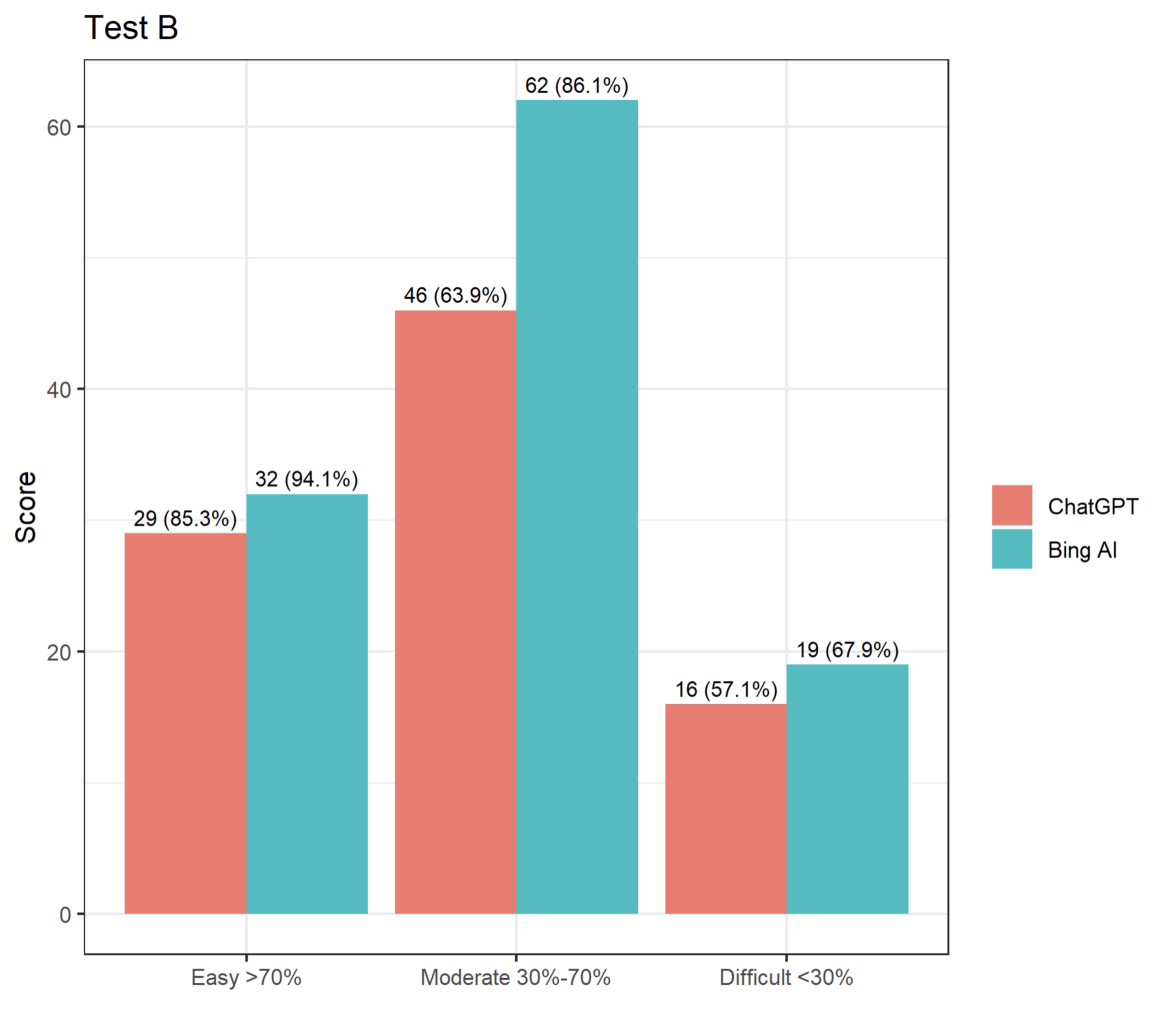
Comparison of answers by ChatGPT and BingAI for Test B.

In test B, BingAI had an accuracy rate of 84.32%, whereas ChatGPT had an accuracy rate of 67.91%, making BingAI approximately 17% more accurate than ChatGPT (Figure 2). BingAI consistently scored higher across all types of questions, with the highest difference observed for moderate questions, where BingAI scored 86.1% (n=62) and ChatGPT scored 63.9% (n=46).

**Figure 3:**
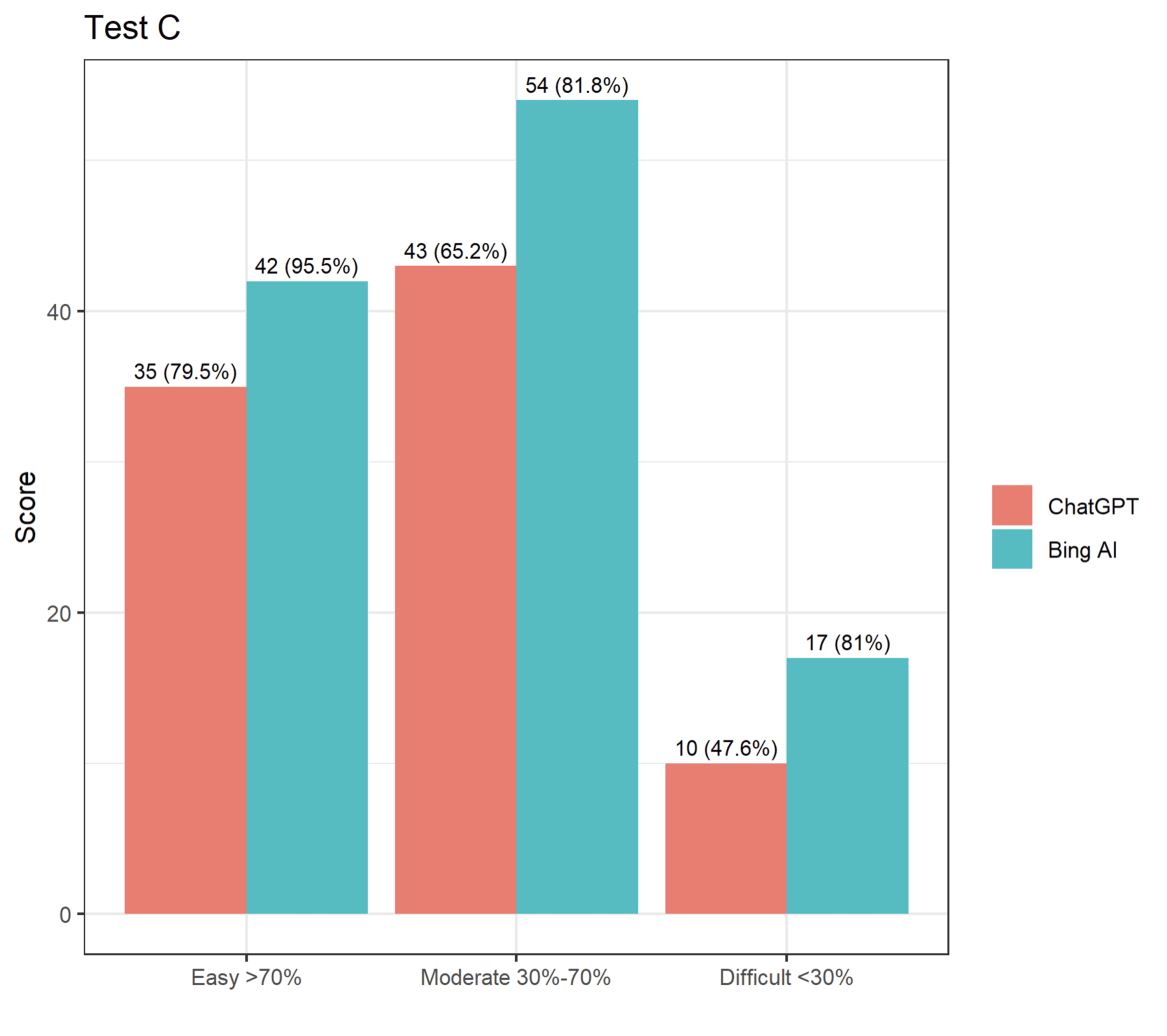
Comparison of answers by ChatGPT and BingAI for Test C.

In test C, BingAI had an accuracy of 86.25%, whereas ChatGPT had an accuracy of 67.17%, making BingAI approximately 19% more accurate than ChatGPT (Figure 3). BingAI consistently scored higher across all types of questions, with the highest difference observed for moderate questions, where BingAI scored 81.8% (n=64) and ChatGPT scored 65.2% (n=43).

From the tables and figures above, it is clear that both AIs could attain a passing score for NEET-PG, and it was also recorded that BingAI could answer questions more correctly in each of the three question banks than ChatGPT. The comparison of answers by ChatGPT and BingAI for Test A, Test B, and Test C is demonstrated in Figures 1-3.

## Discussion

In our study to assess the performance of two AI software, namely, ChatGPT version 3.5 and BingAI Precise mode, in the NEET-PG style questions and exam format, our study found that both these large language models (LLM) were able to successfully answer more than 50% of the questions included in the mock exams. Furthermore, Bing AI Precise mode demonstrated a significantly improved correct response rate compared to ChatGPT-3.5 across various difficulty levels of questions. The difficulty level of the questions was based on how many people who took the mock exam actually got the correct answer to the said question and interestingly LLMs also showed a similar trend as both ChatGPT-3.5 and BingAI Precise mode were able to answer more questions correctly in the easy categorized questions, followed by moderate difficulty and then most difficult questions.

ChatGPT has been used to assess its competency as an LLM in various medical examinations around the world. ChatGPT-3.5 was able to pass all three USMLE steps [9]. It also narrowly passed German Medical Exams M1 and M2, but it failed the Japanese Medical Licensing Exam (JMLE) with a score of 55.1% [10,11]. A reason for this could be because these exams were in their native language of German and Japanese and ChatGPT-3.5 is largely trained in English language. In contrast, ChatGPT-4 passed the JMLE with 87.2% and scored 17% higher than examinees, indicating the advancement in LLM with respect to multiple languages [11]. Similarly, ChatGPT-4 could answer 76.3% of the questions correctly for the United Kingdom Medical Licensing Exam (UKMLA) when taken over an average of three attempts [12]. 

Similar and improving results across various medical licensing examinations with each update of LLMs suggest that it could be used by clinicians and healthcare workers as an aid to their education with proper supervision. Updates to LLMs also suggest that it could be useful as an aid in even non-English speaking countries such as Japan and Germany. Not only helpful to clinicians and healthcare workers, these rapidly advancing LLMs can be useful as a supplementary resource for aspirants preparing for these examinations. These LLMs also have the potential to be integrated into various study resources for such examinations and could change the overall way a person prepares for the said examinations.

Limitations

Our study has some limitations that need to be acknowledged. First, we used a limited number of questions from existing question banks, which may not reflect the full scope and complexity of medical science and questions tested. Second, we did not evaluate the sensitivity of AI software to slight variations in the wording or structure of the questions, which may affect their performance and accuracy. Third, we excluded questions that contained images or tables in the question or answer options, as these types of questions were out of the scope of the AI capability at the time of the study. Fourth, we applied strict inclusion and exclusion criteria to select questions that were answered by all the AI software we compared, which may have introduced some bias and reduced the diversity of the questions. Fifth, we did not account for the updates and improvements that the AI software may undergo over time, which may alter their capabilities and results. Therefore, our findings should be interpreted with caution, and further research is needed to address these limitations.

## Conclusions

The results of our study show that these advancing LLMs such as ChatGPT and BingAI have the ability to answer complex medical questions, which opens the door for them to be used as a study supplementary aid in preparation for these examinations and also as an aid in a clinical setting with proper supervision.

As these LLMs performed similarly to the examinees in terms of answering across various difficulty levels of questions, there is potential for improvement where these LLMs would be able to answer the majority of questions correctly across any difficulty level. There is also potential for these LLMs to interpret and answer questions from images and tables in the future, which would make them an even more excellent resource as a study aid and a tool to be used in the clinical setting to deduce from various radiographic images.
